# Dissolution-and-reduction CVD synthesis of few-layer graphene on ultra-thin nickel film lifted off for mode-locking fiber lasers

**DOI:** 10.1038/srep13689

**Published:** 2015-09-02

**Authors:** Kaung-Jay Peng, Yung-Hsiang Lin, Chung-Lun Wu, Sheng-Fong Lin, Chun-Yu Yang, Shih-Meng Lin, Din-Ping Tsai, Gong-Ru Lin

**Affiliations:** 1 Graduate Institute of Photonics and Optoelectronics, Department of Electrical Engineering, National Taiwan University (NTU), No.1, Sec. 4, Roosevelt Road, Taipei 106, Taiwan, Republic of China; 2 Department of Physics, National Taiwan University (NTU), No.1, Sec. 4, Roosevelt Road, Taipei 106, Taiwan, Republic of China

## Abstract

The *in-situ* dissolution-and-reduction CVD synthesized few-layer graphene on ultra-thin nickel catalyst film is demonstrated at temperature as low as 550 °C, which can be employed to form transmission-type or reflection-type saturable absorber (SA) for mode-locking the erbium-doped fiber lasers (EDFLs). With transmission-type graphene SA, the EDFL shortens its pulsewidth from 483 to 441 fs and broadens its spectral linewidth from 4.2 to 6.1 nm with enlarging the pumping current from 200 to 900 mA. In contrast, the reflection-type SA only compresses the pulsewidth from 875 to 796 fs with corresponding spectral linewidth broadened from 2.2 to 3.3 nm. The reflection-type graphene mode-locker increases twice of its equivalent layer number to cause more insertion loss than the transmission-type one. Nevertheless, the reflection-type based saturable absorber system can generate stabilized soliton-like pulse easier than that of transmission-type system, because the nonlinearity induced self-amplitude modulation depth is simultaneously enlarged when passing through the graphene twice under the retro-reflector design.

Short pulsed fiber laser is the key to explore the ultrafast phenomena or to develop skills in many fields, including biomedical^1^, optical communication^2^, laser surgery^3^, material reactions^4^. The passively mode-locked fiber laser system with compact architecture and high-quality pulse has emerged as the most popular system among candidates nowadays^1^. To start up the mode-locking of fiber lasers, versatile carbon based nano-materials have been applied to serve as saturate absorbers^5^,^6^,^7^,^8^,^9^,^10^,^11^,^12^,^13^,^14^,^15^,^16^,^17^,^18^,^19^,^20^. The carbon nanotubes were demonstrated as the first nano-scale mode locker to generate high-quality pulses^5^,^6^,^7^. However, the high surface energy and aspect ratio of carbon nanotube causes carbon nanotubes easily aggregated and entangled to reduce their surface area and degrades their distribution uniformity. Although the aspect ratio of carbon nanotube could be further reduce by chemical etching^7^, such a strong acid environment with concentrated H_2_SO_4_ and HNO_3_ would form numerous surface defects or destroy the carbon nanotube.

Graphene is a two-dimensional carbon material that could be transferred onto any surface directly. Thus, graphene could overcome the non-uniform distribution and self-aggregation problem happened on carbon nanotube. In addition, graphene exhibits lower threshold intensity for saturate absorption than that of carbon nanotubes to take over other saturable absorbers for the passively mode-locked EDFL^8^,^9^,^10^. Although graphene has many merits, the environmental requirement for synthesizing graphene is relatively strict. Taking the CVD method as an example, high temperature (near 1000 °C) and hydrogen environment are required (One of the research group even think that graphene can hardly be synthesized without “hydrogen” by chemical vapor deposition)^21^. In particular, the oxygen-rare environment is also abundant as the graphene would react with oxygen and formed carbon dioxide.

To get rid of the complicated synthesizing and transferring process, the hydrogen-free and low-temperature plasma enhanced chemical vapor deposition (PECVD) of graphene has emerged^22^. In this work, such a low-temperature and hydrogen-free PECVD synthesized graphene is used as a mode locker in the erbium-doped fiber laser for the first time. In addition, the performances of the graphene saturate absorber in transmission or reflection types for the passively mode-locked EDFL systems are discussed and compared.

## Results

### Atomic-force microscopy, nonlinear transmittance, and Raman scattering spectrum of hydrogen-free low-temperature PECVD synthesized graphene

**Figure 1:**
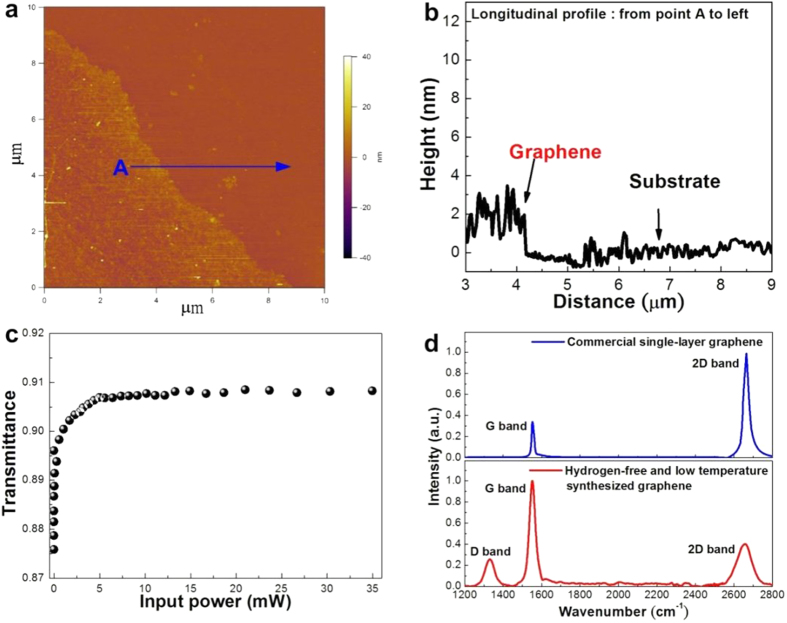
Structural and optical properties of few-layer graphene grown under hydrogen-free and low-temperature PECVD. (**a**) The AFM image and (**b**) the cross-sectional profile scanned from point A of the synthesized few-layer graphene. (**c**) The non-linear transmittance of the synthesized graphene. (**d**) The Raman spectra of commercial single-layer graphene and hydrogen-free synthesized few-layer graphene.

In order to measure the thickness and calculate the layer number of the few-layer graphene, the ultra-thin nickel catalyst film was etched by FeCl_3_ and the lifted graphene was transferred to a smooth Si wafer afterwards. The atomic-force microscopy (AFM) top-view image and cross-sectional profile of few-layer graphene on Si wafer shown in Fig. 1a,b reveal a height difference of 2.5 nm between the Si substrate and the transferred graphene. Considering that the height of monolayer graphene is about 0.33 nm^23^, the layer number of few-layer graphene synthesized by *in-situ* dissolution-and-reduction after the hydrogen-free and low-temperature PECVD growth is roughly estimated as 6 ~ 7 layers. To characterize the saturable absorption feature of the few-layer graphene, the nonlinear transmittance obtained under the pumping with high-peak-power fiber laser (central wavelength at 1570 nm) is shown in Fig. 1c. When the pumping average power increases from 0.008 to 3.23 mW, the transmittance of few-layer graphene nonlinearly increases from 87.5% to 91% with a Δ*T* of 3.5%. The absorption saturates at a pumping power of more than 3.23 mW due to the Pauli blocking effect, where the photons could pass through the optically bleached graphene. The corresponding modulation depth of the few-layer graphene is about 28%, which is already comparable with those of about 30% as obtained from the seven-layer graphene synthesized under high-temperature and hydrogen-rich environment^8^. Such a competitive feature has corroborated the reliability of dissolution-and-reduction syhthesized few-layer graphene grown under hydrogen-free and low-temperature PECVD.

To facilitate the hydrogen-free low-temperature PECVD growth of few-layer graphene, the ultra-thin nickel coated SiO_2_/Si substrate is applied as the catalyst for the dissolution-and-reduction synthesis procedure. Several advantages of nickel which favor the *in-situ* graphene synthesis are addressed below. First, the carbon atoms can still dissolve into the ultra-thin nickel film at relatively low temperature (<500 °C), which facilitates the reduction of few-layer graphene from cooling the nickel matrix after carbon deposition^22^. Second, the layer number of graphene can be precisely controlled with the deposition time under low-temperature and non-hydrogen environment^22^, whereas single-layer graphene can only be obtained by using copper substrate as a catalyst in high-temperature CVD system^24^. In applications, some research groups also observe that it is hardly achieving stabilized soliton-like mode-locking with single- or double-layer graphene^25^. The Raman scattering spectra shown in Fig. 1d consist of two or three typical peaks, in which the D peak around 1328 cm^−1^ is induced by the structural defects in graphene, the G peak at 1580 cm^−1^ certifies the C-C sp^2^ network, and the unique 2D peak at 2760 cm^−1^ denotes the existence of single-layer graphene^26^. The defects may come from the imperfect lattice structure of nickel substrate or the active ions generated by plasma or the sp^3^ carbon bonds^27^. In our case, it is inevitable to generate defects in graphene during the PECVD based dissolution-and-reduction synthesis procedure^28^, and the few-layer graphene lift-off from the nickel catalyst film reveals an I_D_/I_G_ intensity ratio value of about 0.3. The better quality of graphene could be obtained by post annealing on the nickel substrate at 900 °C^29^. Usually, the height of the 2D peak is the simplest way to distinguish the graphene with layer number of less than three. The I_2D_/I_G_ intensity ratio of the graphene after dissolution-and-reduction from ultra-thin nickel film grown by low-temperature hydrogen-free PECVD is about 0.45, indicating that the graphene is not mono-layer (with I_2D_/I_G_ > 1) and bi-layer (with I_2D_/I_G_ = 1). For tri- or more-layer graphene synthesized on ultra-thin nickel catalyst film with hydrogen-free and low-temperature PECVD, the layer number should be further defined by measuring its transmittance^10^, or calculating the dark lines at the edge of graphene from TEM image^30^, or directly measuring its thickness by AFM^31^.

### Transmission-type graphene saturable absorber for passively mode-locked EDFL

**Figure 2:**
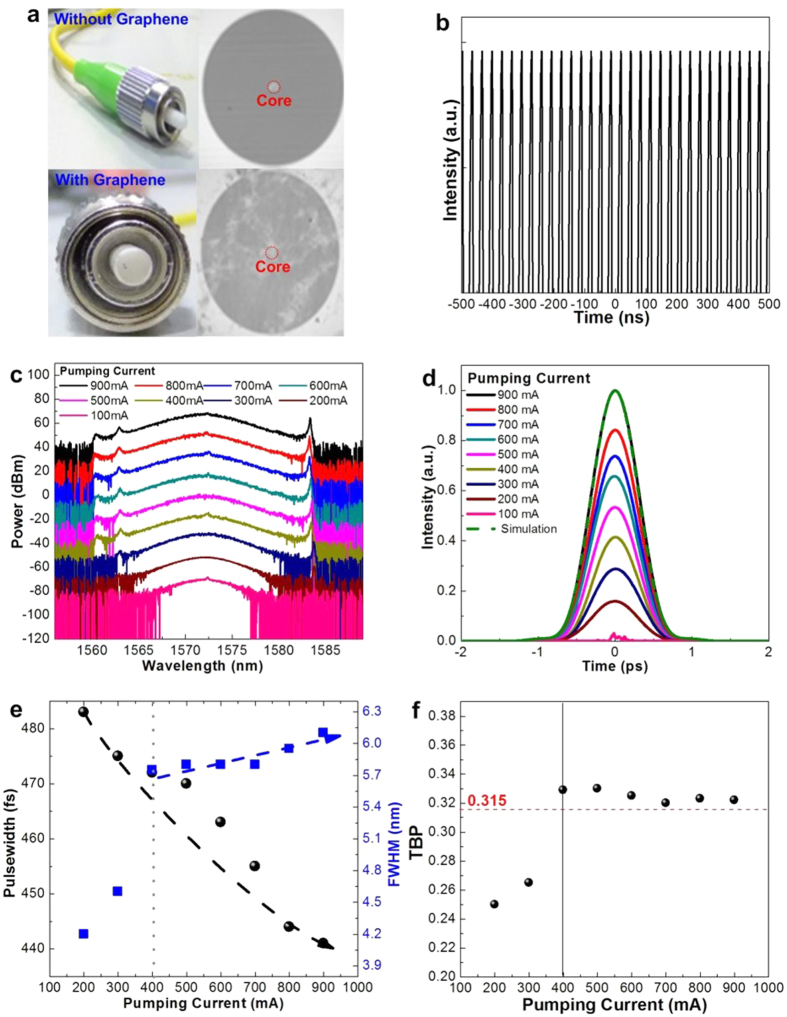
Passively mode-locked EDFL performances by transmission-type graphene saturable absorber. (**a**) The photograph of patchcord end-face with and without hydrogen-free and low temperature synthesized graphene on the surface. (**b**) The oscilloscope trace of passively mode-locked EDFL. (**c**) and (**d**) The optical spectra and autocorrelation traces of transmission-type passively mode-locked EDFL system under different pumping current. (**e**) The varied pulsewidth and FWHM of transmission-type passively mode-locked EDFL system under different pumping current. (**f**) The time-bandwidth products of the transmission-type passively mode-locked EDFL system under different pumping current.

Figure 2a compares the photographs of an SMF connector without and with the few-layer graphene adhered on its end-face, which clearly show the adhered few-layer graphene observed from microscopic images of core and cladding surfaces on the connector end-face. The core area of the connector is remarked by using red-dash circle and the other area at the end-face is the cladding region. According to the photographs, the core area was not covered by any contaminates or residues. To prevent unnecessary scattering and insertion loss caused by those contaminates or residues, each patchcord end-face is checked before experiments so as to minimize the unexpected loss. Although some non-negligible contaminates or residues may attach on the cladding area, the EDFL performances will not be affected accordingly. Under mode-locking with the transmission-type few-layer graphene saturable absorber, the pulse-train depicted in Fig. 2b for the EDFL with a cavity length of 7.43 m reveals the round-trip time and repetition frequency of 35 ns and 28.57 MHz, respectively. The optical spectra of the transmission-type graphene mode-locked EDFL with its central wavelength located around 1572 ± 0.5 nm at different pumping conditions are shown in Fig. 2c. That indicates the pumping power does not affect the location of central wavelength too much in transmission type passively mode-locked EDFL system. The spectral linewidth broadens from 4.2 to 6.1 nm with enlarging the pumping currents from 200 to 900 mA, and the soliton is formed with significant Kelly sidebands at pumping current larger than 400 mA. The higher pumping level is required for obtaining the soliton from the few-layer graphene saturable absorber mode-locked EDFL, which is mainly attributed to the larger linear loss of the 6–7 layer graphene added into the EDFL cavity so as to put off the soliton mode-locking threshold.

In more detail, the autocorrelation traces obtained at different pumping currents beyond the threshold of 100 mA are shown in Fig. 2d, in which the EDFL pulsewidth shortens from 483 to 441 fs (after retrieving with the de-correlation factor of 0.65) by enlarging the pumping current from 200 to 900 mA. The observed pulsewidth is comparable with those obtained in similar system using high-temperature synthesized graphene in hydrogen environment, which corroborates the mode-locking capability of the dissolution-and-reduction synthesized few-layer graphene under hydrogen-free and low-temperature condition. The pulsewidth, linewidth and time-bandwidth products (TBP) as functions of pumping level are plotted in Fig. 2e,f. When reducing the pumping current to 400 mA or lower, the TBP falls below its transform limited value of 0.315 as the peak power of pulse greatly attenuates to fulfill the criterion set by the autocorrelator. As pumping current ranged from 400 to 600 mA, the measured TBP is deviated from 0.315 to indicate the incomplete soliton mode-locking phenomenon. Almost the same temporal and spectral shapes are obtained with nearly transform-limited TBP at pumping current of larger than 700 mA.

### Reflection-type graphene saturable absorber for passively mode-locked EDFL

In comparison, three major differences between the reflection- and transmission-type graphene based passively mode-locked EDFL systems are addressed, the cavity length (due to the aid of circulator), the doubled linear loss and the twice enlarged saturable absorbance (due to retro-reflection in graphene). As the light passes through the graphene layer and reflects by the gold coated end-face, the equivalent layer number increases twice such that the reflection-type graphene based mode-locker inevitably causes more insertion loss than the transmission-type one. Nevertheless, the reflection-type based saturable absorber system was believed to generate stabilized soliton-like pulse easier than that of transmission-type system. That is because the nonlinearity induced self-amplitude modulation depth is simultaneously enlarged passing through the graphene twice under the retro-reflector design. Unfortunately, the mode-locking performance would be slightly degraded with the circulator induced insertion loss, which needs a detailed analysis for comparing both reflection- and transmission-type graphene mode-locked EDFLs.

**Figure 3:**
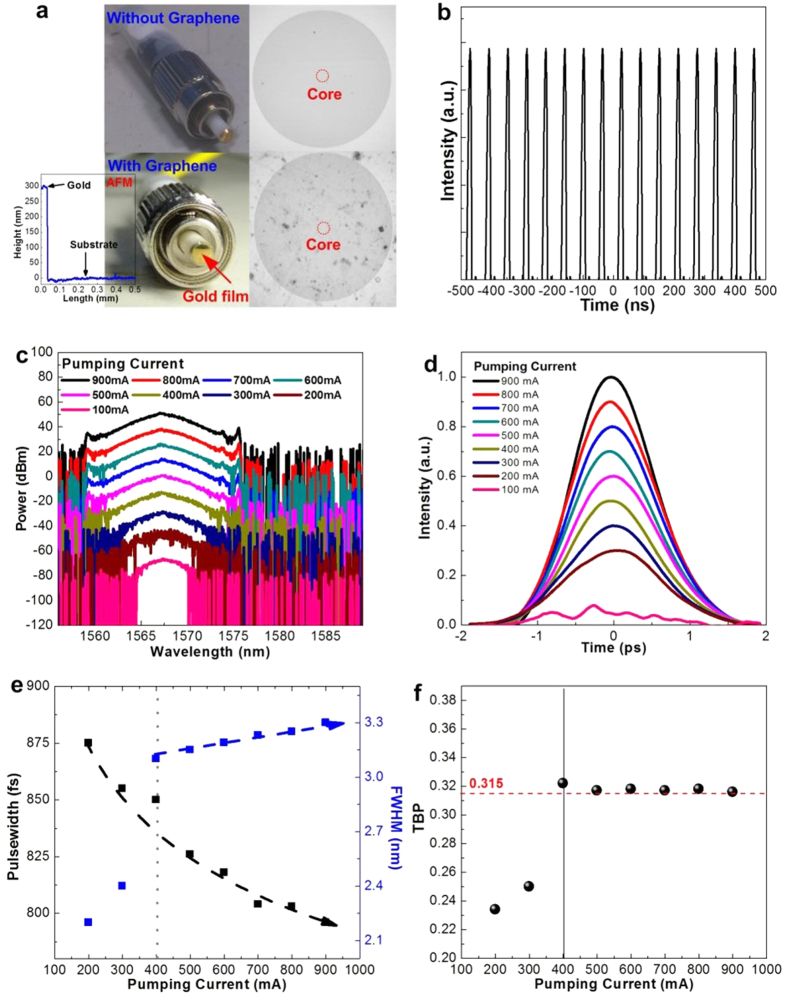
Passively mode-locked EDFL performances by reflection-type graphene saturable absorber. (**a**) The photograph of gold-coated patchcord end-face with and without hydrogen-free and low temperature synthesized graphene on the surface. The inset: AFM image of gold film. (**b**) The oscilloscope trace of passively mode-locked EDFL. (**c**) and (**d**) The optical spectra and autocorrelation traces of reflection-type passively mode-locked EDFL system under different pumping current. (**e**) The varied pulsewidth and FWHM of reflection-type passively mode-locked EDFL system under different pumping current. (**f**) The time-bandwidth products of reflection-type passively mode-locked EDFL system under different pumping current.

To perform the retro-reflection type mode-locker, the Fig. 3a shows the photographs of gold coated SMF connector without and with few-layer graphene. The thickness of gold measured by α-step is shown in the inset of Fig. 3a, which provides a reflectance of up to 99% at wavelength of 1550 nm. The extra loss induced by the circulator about is as large as −1.8 dB. As a result, the reflection-type graphene mode-locked EDFL pulse-train depicted in Fig. 3b exhibits a round-trip time of 60 ns and a repetition frequency of 16.66 MHz as the cavity lengthens to 12.7 m. The optical spectra at different pumping currents shown in Fig. 3c indicate identical central wavelength at 1567 ± 0.5 nm, which is irrelevant to the pumping level as there is no wavelength shift induced beyond lasing or mode-locking. When comparing with the same EDFL system mode-locked by the transmission-type graphene saturable absorber, the 5-nm blue-shift on mode-locking spectrum originates from the enlarged cavity loss mechanism^17^. Owing to the degradation on self-amplitude modulation strength caused by additional loss, the spectral linewidth only broadens from 2.2 to 3.3 nm by enlarging the pumping current from 200 to 900 mA. As expected, the phenomenon of diminished Kelly sideband peak is also accompanied with such a spectral shrinkage under insufficient gain.

From the monitored autocorrelation traces shown in Fig. 3d, the calculated pulsewidth can only compresses from 875 to 796 fs with enlarging the pumping current from 200 to 900 mA, which are about twice broader than those of the pulsewidth obtained from transmission-type system mainly because of extra insertion loss under the same pumping condition. The pulsewidth, linewidth and TBP versus pumping current as plotted in Fig. 3e,f elucidate similar trends like those observed from the transmission-type graphene mode-locked EDFL, indicating that the reflection-type graphene also enables complete soliton mode-locking at pumping power beyond 700 mA. Even though, our observation has corroborated the slightly suppressed self-amplitude modulation as well as degraded mode-locking performance occurs due to the inevitably enlarged intracavity loss of the retro-reflection design for the reflection-type graphene absorber.

Graphene with high crystalline quality can serve as an effective saturable absorber for ultrafast mode-locked lasers due to its excellent optical properties, including ultrafast carrier response time, low saturation intensity, high nonlinear modulation depth and less scattering loss^8^,^10^,^32^. However, structural defects existed in graphene often create scattering centers for phonons and electrons to influence the optotelectronic properties as well as saturation behavior of graphene^27^,^32^. The structural defects would induce nonsaturable absorption and scattering loss, which enlarge the saturation intensity of graphene with a reduced modulation depth to degrade the mode-locking laser performance^20^,^32^. To fabricate a high-quality graphene, the CVD system is a better candidate than other methods^9^,^25^,^32^,^33^ such as mechanical exfoliation^34^,^35^, liquid-phase exfoliation^36^,^37^,^38^, and graphene oxide reduction^39^,^40^, etc. Zhang *et al.* have demonstrated a stable mode-locked EDFL with pulsewidth of 415 fs and pulse energy of 7.3 nJ by using a CVD-grown atomic layer graphene^9^. Huang *et al.* have also utilized the CVD-grown multilayer graphene to produce a stable mode-locked fiber laser with 432-fs pulsewidth^25^. Unfortunately, the growth temperature is needed to be 1000 °C during the synthesis.

With our approach, the desorbed carbon atoms from the Ni film can self-assemble the few-layer graphene with highly layer uniformity. The PECVD synthesis can lower down the growth temperature to a critical value of 550 °C such that the dissolution rate of the decomposed carbon atoms in the Ni film can be minimized. By precisely controlling the growth temperature near the phase transition temperature of Ni, the minimal layer number of graphene can be obtained due to the smallest quantity of carbon atoms desorbed from Ni film. Without hydrogen passivation, the few-layer graphene with suppressed defects can still be obtained under low-temperature growth. Although there is a small amount of defects existed in few-layer graphene to degrade its mode-locking force, the sub-picosecond ultrafast passively mode-locked EDFL pulse can still be generated with the saturable absorption in the *in-situ* dissolution-and-reduction CVD synthesized few-layer graphene.

## Discussion

For the first time, a low-temperature and hydrogen-free PECVD synthesized graphene is used as a mode locker in transmission- and reflection-types for passively mode-locked EDFL systems. A dissolution-and-reduction synthesis facilitates to dissolve carbon atoms into the ultra-thin nickel film at relatively low temperature (~550 °C). Subsequently, the formation of few-layer graphene is observed from cooling the nickel matrix after carbon deposition. The measured layer number of few-layer graphene synthesized by *in-situ* dissolution-and-reduction synthesis is roughly estimated as 6 ~ 7 by AFM. The few-layer graphene obtained under low-temperature and non-hydrogen environment demonstrates a nonlinear transmittance increased from 87.5% to 91% with a Δ*T* of 3.5% and a corresponding modulation depth of 28%. At maximal pumping power, the transmission-type graphene mode-locked EDFL shows a central wavelength of 1572 ± 0.5 nm with a pulsewidth of 441 fs and a spectral linewidth of 6.1 nm. The EDFL with a cavity length of 7.43 m reveals the repetition frequency of 28.57 MHz. The soliton mode-locking is induced by observing the significant Kelly sidebands. In contrast, the reflection-type mode-locker can only generate the passively mode-locked EDFL with a broadened pulsewidth of 796 fs and a shrunk spectral linewidth of 3.3 nm. The layer number the reflection-type graphene based mode-locker is doubled such that the insertion loss becomes twice more than the transmission-type one. The repetition frequency concurrently decreases to 16.66 MHz as the cavity lengthens to 12.7 m. Nevertheless, the reflection-type based saturable absorber system can generate stabilized soliton-like pulse easier than that of transmission-type system, because the nonlinearity induced self-amplitude modulation depth is simultaneously enlarged when passing through the graphene twice under the retro-reflector design.

## Methods

### PECVD synthesis of few-layer graphene and the related transfer process

**Figure 4:**
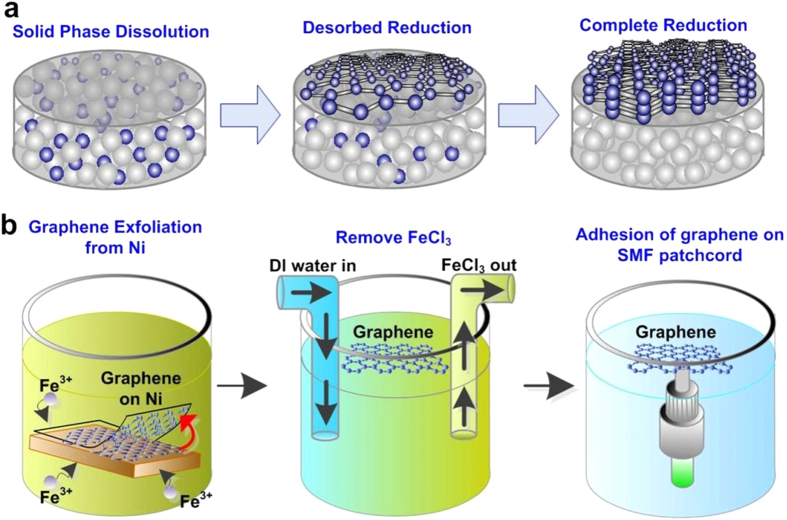
Graphene precipitation from Ni substrate and graphene adhesion on the SMF patchcord. (**a**) The process of graphene precipitation from Ni substrate. (**b**) The transfer process of graphene from Ni substrate to the SMF patchcord.

In experiment, the few-layer graphene at 550 °C is performed with the mixed methane and argon at gas flow rate of 3 and 200 SCCM, respectively. The deposition on 50 nm of Ni substrate by PECVD with the plasma power of 100 W sustains for 100 s. At initial stage, the carbon atoms are gradually dissolved into Ni film at substrate temperature beyond threshold, where the formation of hexagonal carbon ring structure cannot be initiated in the interstices of Ni film. By lowering the substrate temperature, numerous carbon atoms desorb from Ni matrix to form the hexagonal carbon ring structure, and the layer number of graphene on the Ni film is dominated by the amount of desorbed carbon atoms^22^. Figure 4(a) schematically illustrates the formation of few-layer graphene on Ni film. The advantages of such PECVD synthesis are listed below.

### Low-temperature and hydrogen-free PECVD synthesis of few-layer graphene

The advantage of low-temperature growth is to rigidly control the quantity of carbon atoms desorbed from Ni film so as to precisely determine the layer number of self-assembled graphene. Without the need of high-temperature procedure, the precipitation of the dissolved carbon atoms in the Ni film can be reduced. Under the temperature as low as 550 °C (near the critical phase transition temperature of Ni), only a small amount of carbon atoms can be dissolved into the Ni film, so as to prevent the precipitation of abundant carbon atoms after cooling^22^. Such condition can facilitate the synthesis of few-layer graphene. In addition, without the hydrogen passivation for improving the layer uniformity and releasing the defects^21^,^41^, the high-quality few-layer graphene can still be prepared in hydrogen-free environment with the proposed synthesizing method.

### Controllable layer number of *in-situ* dissolution-and-reduction CVD synthesized few-layer graphene

In previous work, the layer number of graphene can be controlled by detuning either the deposition time of argon-diluted methane fluence or the thickness of the evaporated Ni film. For example, when decreasing the deposition time of methane from 600 to 100 s, the graphene layer number can be reduced from 8 to 3 layers^22^.

After hydrogen-free and low-temperature PECVD growth, the synthesized graphene on Ni film is immersed in an aqueous solution of FeCl_3_, where the Fe^3+^ ion can etch the Ni film to make graphene floating upon solution^9^,^42^,^43^. Afterwards, the FeCl_3_ solution is diluted by injecting deionized (DI) water. Eventually, the graphene is attached onto the end-face of a SMF patchcord that is put into the solution of DI water. The transfer procedure is illustrated in Fig. 4(b).

### Passively mode-locked EDFL systems with transmission-type and reflection-type graphene saturable absorbers

**Figure 5:**
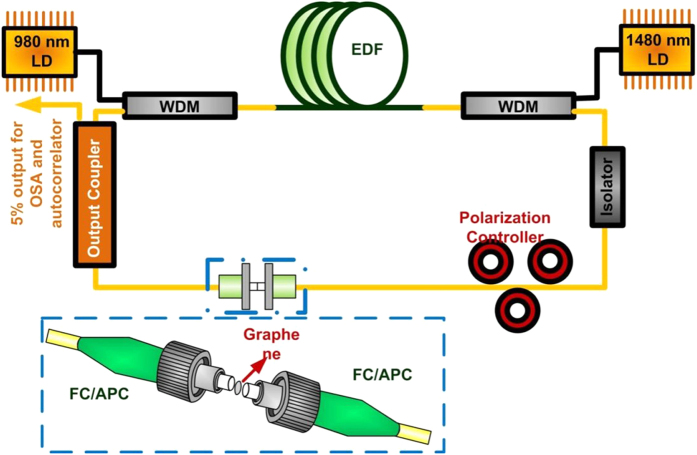
The schematic diagram of transmission-type passively mode locked EDFL. The passively mode-locked EDFL system with the graphene saturable absorber attached on the end-face of SMF patchcord.

These fiber pigtails with few-layer graphene were placed in the passively mode-locked EDFL system, as illustrated in Fig. 5. This system is also known as transmission-type passively mode-locked EDFL used in previous reports^44^. The 2-m long EDF served as the gain medium. The EDF was pumped by laser diodes at 980 and 1480 nm through by wavelength division multiplexer (WDM) couplers. The circulated direction in the EDFL cavity was defined by a polarization independent circulator. The polarization controller was applied to modify the intracavity polarization to optimize the mode locking. A 5% output coupler was used to deliver the EDFL output to optical spectrum analyzer and autocorrelator.

**Figure 6:**
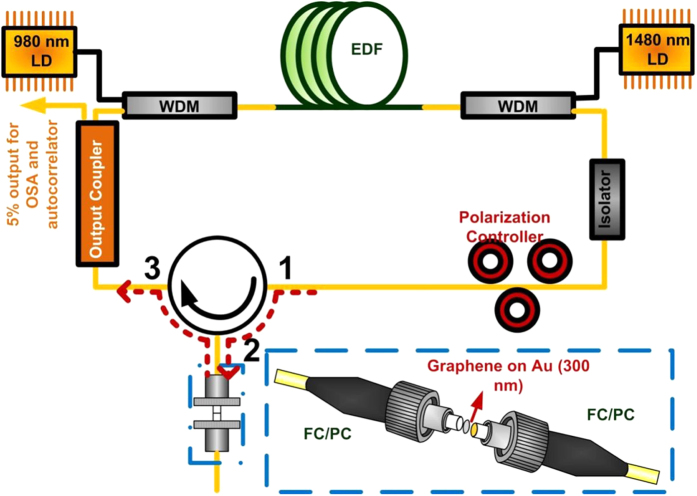
The schematic diagram of reflection-type passively mode locked EDFL. The passively mode-locked EDFL system with the reflection-type graphene saturable absorber attached on the gold film.

In contrast, the second system with a reflection-type few-layer graphene saturable absorber in the passively mode-locked EDFL system is demonstrated as shown in Fig. 6. The other parameters are kept unchanged for the comparison with the transmission-type one. To form the reflection-type saturable absorber, the connector end-face of a SMF patchcord was pre-coated with 300-nm thick gold via the thermal E-gun. After the gold evaporation, the hydrogen-free and low-temperature PECVD synthesized few-layer graphene was transferred onto the surface of gold coated connector end-face and connected to the circulator (port 2). The other ports (1 and 3) of circulator were connected to the system.

**Figure 7:**
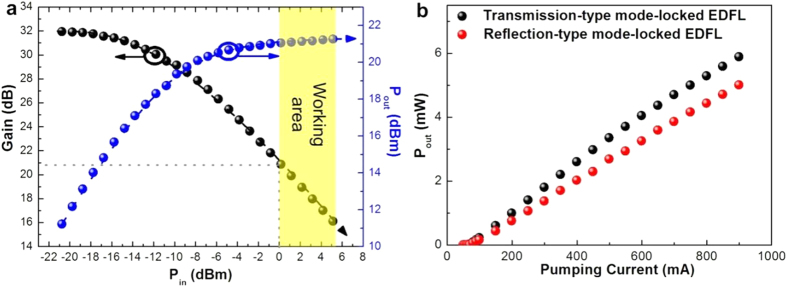
Passively mode-locked EDFL performances. (**a**) The Gain and output power (in dBm) of EDFA versus input power. (**b**) The curves of P_out_ (in mW) vs. pumping currents of the transmission-type and reflection-type graphene saturable absorber mode-locked EDFL systems.

To perform the passive mode-locking at appropriate pumping condition, the intracavity gain of the EDFL was determined under bi-directional pumping regime. The relationship of the output power and the intracavity gain versus input power is shown in Fig. 7(a). The output power is kept linearly increased with almost constant gain of 32 dB under input power of smaller than −10 dBm, which becomes saturated at 21 dBm with corresponding gain decaying to 21 dB under an input power of 0 dBm. The relationship between lasing power and pumping current for both types of passively mode-locked EDFL systems are shown in Fig. 7(b). The maximal lasing power extracted from 5% output coupler of the EDFL system passively mode-locked by transmission-type and reflection-type graphene saturable absorbers are 5.9 and 5 mW, respectively.

## Additional Information

**How to cite this article**: Peng, K.-J. *et al.* Dissolution-and-reduction CVD synthesis of few-layer graphene on ultra-thin nickel film lifted off for mode-locking fiber lasers. *Sci. Rep.* **5**, 13689; doi: 10.1038/srep13689 (2015).

## References

[b1] Keller U. Recent developments in compact ultrafast lasers. Nature 424, 831–838 (2003). 12917697 10.1038/nature01938

[b2] Mollenauer L. F. *et al.* Demonstration of massive wavelength-division multiplexing over transoceanic distances by use of dispersion-managed solitons. Opt. Lett. 25, 704–706 (2000). 18064157 10.1364/ol.25.000704

[b3] Ratkay-Traub I. *et al.* Ultra-short pulse (femtosecond) laser surgery: initial use in LASIK flap creation. Ophthalmol Clin North Am. 14(2), 347–355 (2011). 11406430

[b4] Zewail A. H. Femtochemistry: Recent progess in studies of dynamics and control of reactions and their transition states. J. Phys. Chem. 100, 12701–12724 (1996).

[b5] Yamashita S. *et al.* 5-GHz pulsed fiber Fabry–Pérot laser mode-locked using carbon nanotubes. IEEE Photonics Technol. Lett. 17, 750–752 (2005).

[b6] Wang F. *et al.* Wideband-tuneable, nanotube mode-locked, fibre laser. Nat. Nanotechnol. 3, 738–742 (2008). 19057594 10.1038/nnano.2008.312

[b7] Cheng K. N., Lin Y. H., Yamashita S. & Lin G.-R. Harmonic order-dependent pulsewidth shortening of a passively mode-locked fiber laser with a carbon nanotube saturable absorber. IEEE Photonics J. 4(5), 1542–1552 (2012).

[b8] Bao Q. *et al.* Atomic-layer graphene as a saturable absorber for ultrafast pulsed lasers. Adv. Funct. Mater. 19, 3077–3083 (2009).

[b9] Zhang H., Tang D. Y., Zhao L. M., Bao Q. & Loh K. P. Large energy mode locking of an erbium-doped fiber laser with atomic layer graphene. Opt. Express 17, 17630–17635 (2009). 19907547 10.1364/OE.17.017630

[b10] Sun Z. *et al.* Graphene mode locked ultrafast laser. ACS Nano 4, 803–810 (2010). 20099874 10.1021/nn901703e

[b11] Song Y.-W., Jang S.-Y., Han W.-S. & Bae M.-K. Graphene mode-lockers for fiber lasers functioned with evanescent field interaction. Appl. Phys. Lett. 96, 051122 (2010).

[b12] Zhang H. *et al.* Compact graphene mode-locked wavelength-tunable erbium-doped fiber lasers: from all anomalous dispersion to all normal dispersion. Laser Phys. Lett. 7, 591 (2010).

[b13] Martinez A., Fuse K., Xu B. & Yamashita S. Optical deposition of graphene and carbon nanotubes in a fiber ferrule for passive mode-locked lasing. Opt. Express 18, 22, 23054–23061 (2010). 21164646 10.1364/OE.18.023054

[b14] Lin G.-R. & Lin Y. C. Directly exfoliated and imprinted graphite nano-particle saturable absorber for passive mode-locking erbium-doped fiber laser. Laser Phys. Lett. 8, 880–886 (2011).

[b15] Sobon G., Sotor J. & Abramski K. M. All-polarization maintaining femtosecond Er-doped fiber laser mode-locked by graphene saturable absorber. Laser Phys. Lett. 9(8), 581–586 (2012).

[b16] Jung M. *et al.* Mode-locked pulse generation from an all-fiberized, Tm-Ho-codoped fiber laser incorporating a graphene oxide-deposited side-polished fiber. Opt. Express 21, 20062–20072 (2013). 24105553 10.1364/OE.21.020062

[b17] Lin Y.-H., Chi Y.-C. & Lin G.-R. Nanoscale charcoal powder induced saturable absorption and mode-locking of a low-gain erbium-doped fiber-ring laser. Laser Phys. Lett. 10, 055105 (2013).

[b18] Cui Y. & Liu X. Graphene and nanotube mode-locked fiber laser emitting dissipative and conventional solitons. Opt. Express 21, 18969–18974 (2013). 23938811 10.1364/OE.21.018969

[b19] Ma J. *et al.* Wavelength-versatile graphene-gold film saturable absorber mirror for ultra-broadband mode-locking of bulk lasers. Sci Rep. 4, 5016 (2014). 24853072 10.1038/srep05016 PMC4031483

[b20] Lin Y. H., Yang C. Y., Lin S. F. & Lin G.-R. Triturating versatile carbon materials as saturable absorptive nano powders for ultrafast pulsating of erbium-doped fiber lasers. Opt. Mater. Express 5(2), 236–253 (2015).

[b21] Vlassiouk I. *et al.* Role of hydrogen in chemical vapor deposition growth of large single-crystal graphene. ACS Nano 5, 6069–6076 (2011). 21707037 10.1021/nn201978y

[b22] Peng K.-J. *et al.* Hydrogen-free PECVD growth of few-layer graphene on an ultra-thin nickel film at the threshold dissolution temperature. J. Mater. Chem. C 1, 3862–3870 (2013).

[b23] Gupta A., Chen G. P., Joshi, Tadigadapa S. & Eklund P. C. Raman scattering from high-frequency phonons in supported n-graphene layer films. Nano Lett. 6, 2667–2673 (2006). 17163685 10.1021/nl061420a

[b24] Bhaviripudi S., Jia X., Dresselhaus M. S. & Kong J. Role of kinetic factors in chemical vapor deposition synthesis of uniform large area graphene using copper catalyst. Nano Lett. 10, 4128–4133 (2010). 20812667 10.1021/nl102355e

[b25] Huang P. L. *et al.* Stable mode-locked fiber laser based on CVD fabricated graphene saturable absorber. Opt. Express 20, 2460–2465 (2012). 22330484 10.1364/OE.20.002460

[b26] Ferrari A. C. & Basko D. M. Raman spectroscopy as a versatile tool for studying the properties of graphene. Nat. Nanotechnol. 8, 235–246 (2013). 23552117 10.1038/nnano.2013.46

[b27] Banhart F., Kotakoski J. & Krasheninnikov A. V. Structural defects in graphene. ACS Nano 5, 26–41 (2011). 21090760 10.1021/nn102598m

[b28] Chae S. J. *et al.* Synthesis of large-area graphene layers on poly-nickel substrate by chemical vapor deposition: wrinkle formation. Adv. Mater. 21, 2328–2333 (2009).

[b29] Baraton L. *et al.* Growth of graphene films by plasma enhanced chemical vapour deposition. Proceedings of SPIE 7399, 73990T (2009).

[b30] Qi J. L., Zheng W. T., Zheng X. H., Wang X. & Tian H. W. Relatively low temperature synthesis of graphene by radio frequency plasma enhanced chemical vapor deposition. Appl. Surf. Sci. 257, 6531–6534 (2011).

[b31] Reina A. *et al.* Large area, few-layer graphene films on arbitrary substrates by chemical vapor deposition. Nano Lett. 9, 30–35 (2009). 19046078 10.1021/nl801827v

[b32] Bao Q. *et al.* Monolayer graphene as saturable absorber in mode-locked laser. Nano Res. 4, 297–307 (2011).

[b33] Sobon G. *et al.* Thulium-doped all-fiber laser mode-locked by CVD-graphene/PMMA saturable absorber. Opt. Express 21, 127971–127976 (2013). 10.1364/OE.21.012797 23736498

[b34] Martinez A., Fuse K. & Yamashita S. Mechanical exfoliation of graphene for the passive mode-locking of fiber lasers. Appl. Phys. Lett. 99, 121107 (2011).

[b35] Chang Y. M., Kim H., Lee J. H. & Song Y. W. Multilayered graphene efficiently formed by mechanical exfoliation for nonlinear saturable absorbers in fiber mode-locked lasers. Appl. Phys. Lett. 97, 211102 (2010).

[b36] Bao Q. *et al.* Graphene–polymer nanofiber membrane for ultrafast photonics. Adv. Funct. Mater. 20, 782–791 (2010).

[b37] Lin Y. H., Yang C.-Y., Liou J.-H., Yu C.-P. & Lin G.-R. Using graphene nano-particle embedded in photonic crystal fiber for evanescent wave mode-locking of fiber laser. Opt. Express 21, 16763–16776 (2013). 23938528 10.1364/OE.21.016763

[b38] Bonaccorso F. & Sun Z. Solution processing of graphene, topological insulators and other 2d crystals for ultrafast photonics. Opt. Mater. Express 4, 63–78 (2014).

[b39] Sobon G. *et al.* Graphene oxide vs. reduced graphene oxide as saturable absorbers for Er-doped passively mode-locked fiber laser. Opt. Express 20, 19463–19473 (2012). 23038589 10.1364/OE.20.019463

[b40] He X. *et al.* Passively mode-locked fiber laser based on reduced graphene oxide on microfiber for ultra-wide-band doublet pulse generation. J. Lightwave. Technol. 30, 984–989 (2012).

[b41] Losurdo M., Giangregorio M. M., Capezzuto P. & Bruno G. Graphene CVD growth on copper and nickel: role of hydrogen in kinetics and structure. Phys. Chem. Chem. Phys. 13 , 20836–20843 (2011). 22006173 10.1039/c1cp22347j

[b42] Zhang H., Bao Q., Tang D., Zhao L. & Loh K. P. Large energy soliton erbium-doped fiber laser with a graphene-polymer composite mode locker. Appl. Phys. Lett. 95, 141103 (2009).

[b43] Kim K. S. *et al.* Large-scale pattern growth of graphene films for stretchable transparent electrodes. Nature 457(7230), 706–710 (2009). 19145232 10.1038/nature07719

[b44] Lin Y. H. *et al.* Using n- and p-type Bi_2_Te_3_ topological insulator nanoparticles to enable controlled femtosecond mode-locking of fiber lasers. ACS Photonics 2(4), 481–490 (2015).

